# Evaluation of a novel insoluble fiber source on growth performance and fecal dry matter in nursery pigs using different formulation strategies

**DOI:** 10.1093/tas/txag083

**Published:** 2026-06-20

**Authors:** Julian Arroyave, Mike D Tokach, Jason C Woodworth, Joel M DeRouchey, Jordan T Gebhardt, Katelyn N Gaffield, Robert D Goodband, David Guillou, Noëmie Lemoine

**Affiliations:** Department of Animal Sciences and Industry, College of Agriculture, Kansas State University, Manhattan, KS, 66506-0201, United States; Department of Animal Sciences and Industry, College of Agriculture, Kansas State University, Manhattan, KS, 66506-0201, United States; Department of Animal Sciences and Industry, College of Agriculture, Kansas State University, Manhattan, KS, 66506-0201, United States; Department of Animal Sciences and Industry, College of Agriculture, Kansas State University, Manhattan, KS, 66506-0201, United States; Department of Diagnostic Medicine/Pathobiology, College of Veterinary Medicine, Kansas State University, Manhattan, KS, 66506-0201, United States; Department of Animal Sciences and Industry, College of Agriculture, Kansas State University, Manhattan, KS, 66506-0201, United States; Department of Animal Sciences and Industry, College of Agriculture, Kansas State University, Manhattan, KS, 66506-0201, United States; MixScience, Campus Avril, CS17228, 2, avenue de Ker Lann, Bruz, 35172, France; MixScience, Campus Avril, CS17228, 2, avenue de Ker Lann, Bruz, 35172, France

**Keywords:** Fecal dry matter, fiber, growth performance, nursery pigs, ValoproWin

## Abstract

Three experiments were conducted to evaluate the effects of a novel fiber source (ValoproWin; *VLPW*) composed primarily of insoluble, poorly fermentable fiber on growth performance and fecal dry matter (*DM*) in nursery pigs. In Exp. 1, 300 weanling pigs (initially 5.6 ± 0.47 kg) were allotted to one of six dietary treatments in a 2 × 3 factorial arrangement with main effects of dietary formulation strategy (low acid-binding capacity to a pH of 4; *ABC-4*; vs. conventional) and VLPW feeding duration (0, 10, or 24 d) with a fixed VLPW inclusion of 2.5%. In Exp. 2, 360 weanling pigs (initially 6.3 ± 0.26 kg) were used to evaluate five VLPW levels (0, 1.75, 2.75, 3.75 and 5%) within a low ABC-4 diet and a positive control diet with no added VLPW and the ABC-4 was not considered (conventional). In Exp. 3, 335 weanling pigs (initially 5.6 ± 0.87 kg) were used in a 2 × 2 + 1 factorial evaluating VLPW addition (2.5 or 5%) and formulation method (diluted or adjusted), with a control diet without VLPW. Across all three experiments, pigs were fed experimental diets during the first two dietary phases (approximately from weaning at 21 d of age to d 24 postweaning), followed by a common phase 3 diet. In experiments 1 and 2, no nutrient adjustments were made to the diet when VLPW was added, which resulted in nutrient dilution proportional to the VLPW level. In Exp. 1, VLPW feeding duration did not affect growth performance, whereas in Exp. 2, increasing VLPW tended (quadratic, *P* = 0.067) to decrease overall average daily gain (***ADG***). In Exp. 3, pigs fed diets with 5% of VLPW during the experimental period tended (*P* = 0.057) to have decreased gain-to-feed ratio compared with those fed diets with 2.5% VLPW. Across all experiments, although low ABC-4 diets or diet dilution affected growth performance during the experimental period, no overall differences were observed. In Exp. 1, added VLPW increased fecal DM at d 10 (*P* = 0.019). In Exp. 2, fecal DM increased (linear, *P* < 0.005) on d 10 and 24 as VLPW increased. Similarly, in Exp. 3, increasing VLPW increased (linear, *P* < 0.001) fecal DM on d 10, regardless of formulation method. In conclusion, VLPW increased fecal DM in weanling pigs but had no effects on overall growth performance across multiple dietary strategies and inclusion levels.

## Teaser

The use of a novel fiber source during the first two dietary phases consistently increased fecal dry matter; however, these benefits did not translate into improvements in growth performance of nursery pigs.

## Introduction

Dietary fiber has traditionally been defined as a carbohydrate consisting of three or more monomeric units (including non-digestible oligosaccharides) that resist hydrolysis by endogenous enzymes in the gastrointestinal tract of monogastric animals (Howlett et al. 2010; Jones 2014). Consequently, fiber has historically been considered an antinutritional factor in swine nutrition, particularly in nursery diets, due to its association with reduced nutrient digestibility and growth performance in young pigs (Berrocoso et al. 2015; Jha and Berrocoso 2015).

Advancements in analytical techniques and an improved understanding of the nutritional role of fiber has led to more precise classifications of dietary fiber based on its physicochemical properties, particularly fermentability and solubility (Bach Knudsen 2001; Montagne et al. 2003). Fiber is now commonly categorized as either soluble or insoluble, depending on its solubility in water (Lattimer and Haub 2010). Soluble fiber can serve as a fermentable substrate for beneficial bacteria in the gastrointestinal tract (Hu et al. 2023), while insoluble fiber helps to regulate intestinal transit and reduce the colonization of pathogenic microbes (Dorado-Montenegro et al. 2023; Li et al. 2020). Despite these benefits, the effects of dietary fiber on growth performance remain inconsistent (Grześkowiak et al. 2022). Variations in outcomes are likely influenced by fiber type, inclusion level, and interaction with other dietary components (Lv et al. 2022; Zhao et al. 2023).

ValoproWin (*VLPW*; MiXscience, Bruz, France) is a novel fiber ingredient specifically designed for nursery pig diets. It is mainly composed of a purified source of fiber, oat hulls, and yeast autolysate. ValoproWin has a water-holding capacity (WHC) of 4.3 mL/g and an average particle size of 321 ± 1.96 µm. Fiber composition, analyzed using the sequential fiber fractionation procedure of Van Soest et al. (1991), consists of 75.3% neutral detergent fiber (NDF), 61.3% acid detergent fiber (ADF), and 19.4% lignin, indicating a high concentration of structural carbohydrates. The composition suggests a predominance of insoluble, poorly fermentable fiber components, which may contribute to increased intestinal motility and enhanced gut barrier function in weaned pigs (Jha and Berrocoso 2015; Montagne et al. 2003). The inclusion of yeast autolysate and hemicellulose contained in the NDF fraction may also provide soluble fiber and functional compounds such as β-glucans and mannans, which have been associated with immune modulation and reduced pathogen colonization through the binding and sequestration of pathogenic microorganisms in the gastrointestinal tract (Choi and Kim 2023).

Although two conference abstracts describing prototypes of VLPW with emphasis on fiber components have been presented (Lemoine et al. 2018, 2022), no published studies have evaluated the finalized VLPW formulation effects on nursery pig growth performance or fecal dry matter (*DM*). Therefore, the objectives of the present studies were to determine the influence of VLPW on growth performance and fecal DM of nursery pigs. Within three experiments, sub-objectives were to determine how the response was affected by: (1) VLPW feeding duration and dietary acid binding capacity at a pH of 4 (ABC-4); (2) VLPW inclusion level; and (3) potential interactions between VLPW inclusion level and dietary formulation strategies (nutrient dilution vs. nutrient adjustment).

## Materials and methods

### General

The Kansas State University Institutional Animal Care and Use Committee approved the protocols used in these experiments (IACUC # 4485.54, 4943, and 4942). Experiment (*Exp*.) 1 was conducted between November and December 2023 in two independent rooms within the same barn located at the Kansas State University Swine Teaching Center in Manhattan, KS. Each pen (1.2 × 1.5 m) provided approximately 0.36 m^2^ per pig and was equipped with a 4-hole dry feeder and nipple waterer. Experiment 2 was conducted between August and September 2024 in two identical barns located at the Kansas State University Segregated Early Weaning facility in Manhattan, KS. Each pen (1.2 × 1.5 m) provided approximately 0.36 m^2^ per pig and was equipped with a 4-hole dry feeder and a cup drinker. Experiment 3 was conducted between June and July 2025 in a single barn located at the Kansas State University Swine Teaching Center in Manhattan, KS. Each pen (1.5 × 1.5 m) provided approximately 0.45 m^2^ per pig and was equipped with a 6-hole dry feeder and nipple waterer. All facilities were mechanically ventilated. Room temperatures were set at 32.2°C during the first 10 d after weaning and then gradually decreased by 0.28°C daily until the set point reached 25°C.

### Animals and diets

In Exp. 1, a total of 300 pigs (gilts and barrows, Line 241 × 600, DNA, Columbus, NE) initially weighing 5.6 ± 0.47 kg were used in a 42-d study. Pigs were weaned at approximately 19 d of age and divided into five body weight (BW) blocks, each containing 20% of the pigs. Pigs were then randomly assigned to pens within BW block and pens (experimental units) were allotted to one of six dietary treatments using a generalized complete block design. Each pen had 5 pigs, and there were 10 pens per dietary treatment. The two identical rooms had an equal representation of dietary treatments and BW blocks. Pigs were fed experimental diets for the first two phases, lasting 10 and 14 d, respectively. All pigs were then fed a common phase 3 diet from d 24 to 42. Treatments were arranged in a 2 × 3 factorial with the main effects of formulation strategy (low ABC-4 or conventional) and VLPW feeding duration immediately after weaning (0, 10, or 24 d). Low ABC-4 diets were formulated to 200 and 250 meq/kg from d 0 to 10 and d 10 to 24, respectively, without pharmacological levels of Zn from ZnO (Table 1). Pure lactose and specialty soy protein concentrate (AX3 Digest, Protekta, Newport Beach, CA) were used in these diets as lactose and protein concentrate sources, respectively, along with fumaric and formic acid to lower pH. Other treatment diets were formulated without considering ABC-4 values (conventional) that were 493 and 470 meq/kg and 2990 and 1910 ppm of Zn from d 0 to 10 and 10 to 24, respectively. Spray-dried whey and enzymatically treated soybean meal (HP300, Hamlet Protein, Findlay, OH) were used as lactose and specialty soy sources, respectively. ValoproWin was added at 2.5% of the diet (manufacturer’s recommendation), without any formulation adjustments resulting in slightly different chemical composition between diets. By design, this resulted in a dilution of approximately 2.5% in all nutrients compared to the control diet. Pigs and feeders were weighed on d 0, 10, 24, and 42 to determine average daily gain (*ADG*), average daily feed intake (*ADFI*), and gain-to-feed ratio (*G:F*) ratio. Fecal samples were collected via rectal palpation from the same three pigs per pen on d 10 and 24 of the study to determine fecal DM.

**Table 1:** Diet composition, Exp. 1 (as-fed basis).^a^

	Phase 1				Phase 2				Phase 3
	Low ABC-4		Conventional		Low ABC-4		Conventional		
* ZnO:*	No	No	Yes	Yes	No	No	Yes	Yes	
*Item ValoproWin:*	No	Yes	No	Yes	No	Yes	No	Yes	
***Ingredient, %***									
*** Corn***	50.76	49.49	48.23	47.03	57.17	55.74	55.17	53.79	68.06
*** Soybean meal, 47.7% CP***	16.30	15.89	16.27	15.86	22.40	21.84	26.06	25.41	28.12
*** Crystalline lactose***	15.00	14.63	–	–	7.50	7.31	–	–	–
*** Spray-dried whey powder***	–	–	20.85	20.33	–	–	10.40	10.14	–
*** Spray-dried bovine plasma***	2.50	2.44	2.50	2.44	–	–	–	–	–
*** Plant protein concentrate^b^***	9.38	9.14	–	–	7.50	7.31	–	–	–
*** Specialty soybean meal^c^***	–	–	7.75	7.56	–	–	4.00	3.90	–
*** VLPW^d^***	–	2.50	–	2.50	–	2.50	–	2.50	–
*** Corn oil***	2.00	1.95	2.00	1.95	1.00	0.98	1.00	0.98	–
*** Monocalcium phosphate, 21% P***	0.93	0.90	0.20	0.20	1.00	0.98	0.60	0.59	0.85
*** Limestone***	0.35	0.34	0.33	0.32	0.51	0.50	0.52	0.51	0.75
*** Salt***	0.65	0.63	0.28	0.27	0.70	0.68	0.55	0.54	0.60
*** L-Lys-HCl***	0.44	0.43	0.36	0.35	0.49	0.47	0.45	0.44	0.55
*** DL-Met***	0.19	0.18	0.16	0.16	0.19	0.19	0.19	0.19	0.21
*** L-Thr***	0.19	0.19	0.14	0.13	0.21	0.20	0.19	0.19	0.23
*** L-Trp***	0.05	0.05	0.03	0.03	0.05	0.05	0.04	0.04	0.05
*** L-Val***	0.08	0.08	0.07	0.06	0.10	0.10	0.12	0.11	0.16
*** Trace mineral premix^e^***	0.15	0.15	0.15	0.15	0.15	0.15	0.15	0.15	0.15
*** Vitamin premix^f^***	0.25	0.24	0.25	0.24	0.25	0.24	0.25	0.24	0.25
*** Phytase^g^***	0.06	0.05	0.06	0.05	0.06	0.05	0.06	0.05	0.03
*** Zinc oxide***	–	–	0.40	0.39	–	–	0.25	0.24	–
*** Fumaric acid^h^***	0.38	0.37	–	–	0.38	0.37	–	–	–
*** Formic acid^i^***	0.36	0.35	–	–	0.36	0.35	–	–	–
***Total***	100.00	100.00	100.00	100.00	100.00	100.00	100.00	100.00	100.00
*** Calculated analysis***									
***Standardized ileal digestible amino acids, %***									
*** Lys***	1.36	1.33	1.36	1.33	1.35	1.32	1.35	1.32	1.30
*** Ile:Lys***	56	56	58	58	58	58	58	58	53
*** Leu:Lys***	116	116	118	118	116	116	115	115	113
*** Met:Lys***	34	34	32	32	35	35	35	35	36
*** Met & Cys:Lys***	56	56	56	56	56	56	56	56	57
*** Thr:Lys***	63	63	63	63	63	63	63	63	63
*** Trp:Lys***	20	20	20	20	20	20	20	20	19
*** Val:Lys***	70	70	70	70	70	70	70	70	70
*** His:Lys***	36	36	35	35	36	36	36	36	35
***Net energy, Mcal/kg***	2.60	2.53	2.61	2.54	2.50	2.44	2.50	2.44	2.45
***SID Lys:NE, g/Mcal***	5.23	5.25	5.21	5.23	5.40	5.41	5.40	5.41	5.31
***CP, %***	21.1	20.5	21.0	20.5	21.3	20.8	21.2	20.7	20.0
***Ca, %***	0.50	0.49	0.49	0.48	0.59	0.57	0.59	0.58	0.64
***P, %***	0.53	0.51	0.52	0.50	0.56	0.56	0.56	0.56	0.56
***STTD P, %^j^***	0.46	0.45	0.46	0.45	0.46	0.45	0.46	0.45	0.43
***Crude fiber, %***	1.64	2.97	1.88	3.11	2.00	3.33	2.26	3.57	2.44
***NDF, %^k^***	5.96	7.70	6.09	6.52	7.05	8.75	7.35	9.05	8.51
***ADF, %^l^***	2.32	3.80	2.53	4.17	2.83	4.29	3.11	4.57	3.45
***Lignin, %***	0.34	2.23	0.33	2.22	0.43	2.32	0.46	2.35	0.53
***Analyzed composition***									
*** Crude protein, %***	19.1	19.2	20.0	19.7	19.8	19.6	20.6	20.6	18.1
*** NDF, %***	8.0	8.1	5.8	7.6	7.0	9.8	7.4	8.3	9.09
*** ADF, %***	2.9	4.0	2.3	3.7	2.8	4.4	2.9	4.1	3.1
*** ADL, %^m^***	0.08	0.63	0.10	0.64	0.08	0.62	0.08	0.62	0.04
*** ABC-4, meq/kg^n^***	206	215	510	495	261	253	488	453	—

a Phases 1, 2, and 3 were fed for 10, 14, and 18 d, respectively. Diets were formulated to meet or exceed the nutrient requirements recommended by the NRC (2012) for pigs with average body weights of 6, 9, and 18 kg for phases 1, 2, and 3, respectively.

b AX3 Digest (Proteka, Newport Beach, CA).

c HP 300 (Hamlet protein, Findlay, OH).

d ValoproWin (Mixscience, Bruz, France).

e Provided per kilogram of feed: 4134 IU of vitamin A; 1653 IU of vitamin D; 44 IU of vitamin E; 3.31 mg of vitamin K3; 33 µg of vitamin B_12_; 8 mg of riboflavin; 28 mg of pantothenic acid; 50 mg of niacin.

f Provided per kilogram of feed: 110 mg of Zn as zinc sulfate; 17 mg of Cu as copper sulfate; 33 mg of Mn as manganese oxide; 110 mg of Fe as ferrous sulfate; 0.30 mg of I as ethylenediamine dihydriodide; 0.30 mg of Se as sodium selenite.

g Ronozyme Hiphos 2700 (dsm-firmenich, Parsippany, NJ) provided 1486 FYT/kg for phases 1 and 2 and 811 FYT/kg in phase 3 with an assumed release of 0.14% STTD P in phases 1 and 2 and 0.12% STTD P in phase 3.

h Primary Products Ingredients Americas LLC, Decatur, IL.

i pHormasil Na (Hawkins, Roseville, MN).

j Standardized total tract digestible phosphorus.

k Neutral detergent fiber.

l Acid detergent fiber.

m Acid detergent lignin.

n Acid binding capacity to a pH of 4.

In Exp. 2, a total of 360 barrows (Line 200 × 400, DNA, Columbus, NE) initially 6.3 ± 0.26 kg were used in a 42-d study. The pigs were weaned at approximately 21 d of age and divided into six BW blocks. The pigs were then randomly assigned to pens within the BW category and pens were allotted to one of six dietary treatments using a generalized complete block design. Each pen had five pigs and there were 12 pens per treatment. The two identical barns had an equal representation of dietary treatments and BW categories. Pigs were fed experimental diets for the first two dietary phases, lasting 10 and 14 d, respectively. From d 24 to 42 of the experiment, all pigs were fed a common phase 3 diet. Dietary treatments included a low ABC-4 diet with incremental levels of VLPW (0, 1.75, 2.50, 3.75, or 5% of the diet), as well as a positive control diet without VLPW, where the ABC-4 capacity of the diet was not controlled (conventional) and pharmacological levels of Zn from ZnO were added during phases 1 and 2 (Table 2). The formulation strategy was the same as Exp. 1, differing only in the specialty soy ingredients, where specialty soybean meal (AX3 Digest; Proteka, Newport Beach, CA) was replaced with microbial enhanced protein (ME-Pro, Prairie Aquatech, Brookings, SD). For VLPW diet preparation, a large batch of basal low ABC-4 phase 1 and 2 diets were mixed and placed in separate bins. A portion of the basal diet was then blended with VLPW to create experimental diets, without adjusting the nutrient levels, resulting in a reduction in the chemical composition of the diets as VLPW increased. Pigs and feeders were weighed on d 10, 24, and 42 to determine ADG, ADFI, and G:F. Fecal samples were collected via rectal palpation from the same three pigs per pen on d 10 and 24 of the study.

**Table 2:** Diet composition, Exp. 2 (as-fed basis).^a^

	**Phase 1**		**Phase 2**		Phase 3
Item	Low ABC-4	Conventional	Low ABC-4	Conventional	—
***Ingredients, %***					
*** Corn***	50.56	48.88	55.97	55.26	68.06
*** Soybean meal, 47.7% CP***	17.34	17.35	25.35	25.35	28.12
*** Crystalline lactose***	15.00	—	7.50	—	—
*** Spray-dried whey powder***	—	20.85	—	10.40	—
*** Spray-dried bovine plasma***	3.25	3.25	—	—	—
*** Fermented soybean meal^b^***	7.50	—	5.50	0.00	—
*** Specialty soybean meal^c^***	—	5.26	—	4.62	—
*** Corn oil***	2.00	2.00	1.00	1.00	—
*** Monocalcium phosphate, 21% P***	0.91	0.20	0.97	0.60	0.85
*** Limestone***	0.33	0.33	0.48	0.52	0.75
*** Salt***	0.77	0.28	0.80	0.55	0.60
*** L-Lys-HCl***	0.44	0.36	0.49	0.45	0.55
*** DL-Met***	0.24	0.16	0.24	0.19	0.21
*** L-Thr***	0.18	0.14	0.21	0.19	0.23
*** L-Trp***	0.04	0.04	0.05	0.05	0.05
*** L-Val***	0.09	0.07	0.11	0.12	0.16
*** Vitamin premix^d^***	0.25	0.25	0.25	0.25	0.25
*** Trace mineral premix^e^***	0.15	0.15	0.15	0.15	0.15
*** Phytase^f^***	0.06	0.06	0.06	0.06	0.03
*** Zinc oxide***	—	0.40	—	0.25	—
*** Fumaric acid^g^***	0.50	—	0.50	—	—
*** Formic acid^h^***	0.40	—	0.40	—	—
***TOTAL***	100.00	100.00	100.00	100.00	100.00
***Calculated analysis***					
***Standardized ileal digestible amino acids, %***					
*** Lys***	1.36	1.36	1.35	1.35	1.30
*** Ile:Lys***	56	57	58	58	53
*** Leu:Lys***	115	118	115	115	113
*** Met:Lys***	37	31	39	35	36
*** Met & Cys:Lys***	56	56	56	56	57
*** Thr:Lys***	63	63	63	63	63
*** Trp:Lys***	20	21	21	21	19
*** Val:Lys***	70	70	70	70	70
*** His:Lys***	36	35	36	36	35
***NE, Mcal/kg***	2.59	2.61	2.48	2.50	2.45
***SID Lys:NE, g/Mcal***	5.26	5.21	5.44	5.40	5.31
***CP, %***	21.05	20.80	21.46	21.24	19.98
***Lactose, %***	15.00	15.00	7.50	7.50	− - -
***Ca, %***	0.49	0.49	0.57	0.59	0.64
***P, %***	0.52	0.52	0.55	0.56	0.56
***STTD P, %^i^***	0.47	0.46	0.46	0.46	0.43
***Crude fiber, %***	2.20	1.87	2.48	2.27	2.44
***NDF, %***	7.49	6.36	8.25	7.54	8.51
***ADF, %***	3.10	2.63	3.49	3.20	3.45
***Lignin, %***	0.42	0.35	0.51	0.46	0.53

a Phases 1, 2, and 3 were fed for 10, 14, and 18 d, respectively. Diets were formulated to meet or exceed the nutrient requirements recommended by the NRC (2012) for pigs with average body weights of 6, 9, and 18 kg for phases 1, 2, and 3, respectively. ValoproWin was mixed with the low ABC-4 basal diet at 0, 1.75, 2.50, 3.75, and 5.00% of the diet with no other adjustment in the formulation made.

b ME-Pro (Prairie Aquatech, Brookings, SD).

c HP 300 (Hamlet protein, Findlay, OH).

d Provided per kilogram of feed: 4134 IU of vitamin A; 1653 IU of vitamin D; 44 IU of vitamin E; 3.31 mg of vitamin K3; 33 µg of vitamin B_12_; 8 mg of riboflavin; 28 mg of pantothenic acid; 50 mg of niacin.

e Provided per kilogram of feed: 110 mg of Zn as zinc sulfate; 17 mg of Cu as copper sulfate; 33 mg of Mn as manganese oxide; 110 mg of Fe as ferrous sulfate; 0.30 mg of I as ethylenediamine dihydriodide; 0.30 mg of Se as sodium selenite.

f Ronozyme Hiphos 2700 (dsm-firmenich, Parsippany, NJ) provided 1486 FYT/kg for phases 1 and 2 and 811 FYT/kg in phase 3 with an assumed release of 0.14% STTD P in phases 1 and 2 and 0.12% STTD P in phase 3.

g Primary Products Ingredients Americas LLC, Decatur, IL.

h pHormasil Na (Hawkins, Roseville, MN).

i Standardized total tract digestible phosphorus.

In Exp. 3, a total of 335 pigs (gilts and barrows, Line 241 × 600, DNA, Columbus, NE) initially weighing 5.6 ± 0.87 kg were used in a 45-d study. The pigs were weaned at approximately 19 d of age and divided into two BW categories. Pigs were then randomly assigned to pens within the BW categories, and pens were allotted to one of five dietary treatments using a generalized complete block design. Each pen had four or five pigs, and there were 14 pens per treatment. Pigs were fed experimental diets for the first two dietary phases, lasting 10 and 13 d, respectively. From d 23 to 45 of the experiment, all pigs were fed a common phase 3 diet. Dietary treatments were arranged in a 2 × 2 + 1 factorial, with the main effects of VLPW (2.5 or 5%) and formulation method (diluted or adjusted). An additional control treatment, which contained no VLPW, was used for comparison. In the diluted diets, VLPW was added at the expense of the complete diet without further adjustments to the formulation, resulting in nutrient dilution proportional to the VLPW inclusion. In contrast, adjusted diets were reformulated to maintain a similar nutrient composition to the control diet, regardless of VLPW inclusion level (Table 3). Thus, adjusted diets contained increasing soybean meal, soy oil, and monocalcium phosphate to maintain similar amino acid, energy, and phosphorus levels across diets as VLPW increased. Pigs and feeders were weighed on d 10, 23, and 45 to determine ADG, ADFI, and G:F. Fecal samples were collected via rectal palpation from the same three pigs per pen on d 10 and 23 of the study.

**Table 3:** Diet composition, Exp. 3 (as-fed basis).^a^

	Phase 1					Phase 2					Phase 3
		Diluted		**Adjusted**			Diluted		**Adjusted**		—
Item ValoproWin, %:	0	2.5	5	2.5	5	0	2.5	5	2.5	5	—
***Ingredient, %***											
*** Corn***	50.54	49.27	48.01	46.59	42.43	55.97	54.57	53.17	51.77	47.65	68.05
*** Soybean meal, 47.7% CP***	17.35	16.91	16.48	17.67	18.11	25.35	24.72	24.09	25.94	26.37	28.12
*** Crystalline lactose***	15.00	14.63	14.25	15.00	15.00	7.50	7.31	7.13	7.50	7.50	—
*** Fermented soybean meal^b^***	7.50	7.31	7.13	7.50	7.50	5.50	5.36	5.23	5.50	5.50	—
*** Spray-dried bovine plasma***	3.25	3.17	3.09	3.25	3.25	—	—	—	—	—	—
*** VLPW^c^***	—	2.50	5.00	2.50	5.00	—	2.50	5.00	2.50	5.00	—
*** Soybean oil***	2.00	1.95	1.90	3.09	4.28	1.00	0.98	0.95	2.15	3.28	—
*** Monocalcium phosphate, 21% P***	0.91	0.89	0.87	0.95	0.96	0.97	0.94	0.92	0.97	1.00	0.85
*** Limestone***	0.33	0.32	0.31	0.31	0.30	0.48	0.46	0.45	0.45	0.43	0.75
*** Salt***	0.77	0.75	0.73	0.77	0.78	0.80	0.78	0.76	0.81	0.81	0.6
*** L-Lys-HCl***	0.44	0.43	0.42	0.43	0.43	0.49	0.47	0.46	0.47	0.46	0.55
*** DL-Met***	0.25	0.24	0.24	0.25	0.26	0.23	0.22	0.22	0.24	0.26	0.21
*** L-Thr***	0.18	0.18	0.17	0.19	0.20	0.21	0.20	0.19	0.21	0.22	0.23
*** L-Trp***	0.04	0.04	0.04	0.04	0.04	0.05	0.05	0.05	0.05	0.05	0.05
*** L-Val***	0.09	0.08	0.08	0.10	0.10	0.11	0.10	0.10	0.11	0.12	0.16
*** Vitamin premix^d^***	0.25	0.24	0.24	0.25	0.25	0.25	0.24	0.24	0.25	0.25	0.25
*** Trace mineral premix^e^***	0.15	0.15	0.14	0.15	0.15	0.15	0.15	0.14	0.15	0.15	0.15
*** Phytase^f^***	0.06	0.06	0.06	0.06	0.06	0.06	0.06	0.06	0.06	0.06	0.03
*** Fumaric acid^g^***	0.50	0.49	0.48	0.50	0.50	0.50	0.49	0.48	0.50	0.50	—
*** Formic acid^h^***	0.40	0.39	0.38	0.40	0.40	0.40	0.39	0.38	0.40	0.40	—
***TOTAL***	100.00	100.00	100.00	100.00	100.00	100.00	100.00	100.00	100.00	100.00	100.00
***Calculated analysis***											
***Standardized ileal digestible amino acids, %***											
*** Lys***	1.36	1.33	1.30	1.36	1.36	1.35	1.32	1.29	1.35	1.35	1.30
*** Ile:Lys***	56	56	56	56	56	58	58	58	59	59	53
*** Leu:Lys***	115	115	115	113	112	115	115	115	114	113	113
*** Met:Lys***	38	38	38	38	38	38	38	38	38	39	36
*** Met & Cys:Lys***	56	56	56	56	56	55	55	55	55	56	57
*** Thr:Lys***	63	63	63	63	64	63	63	63	63	64	63
*** Trp:Lys***	20	21	21	21	20	20	20	20	20	21	19
*** Val:Lys***	70	70	70	70	70	70	70	70	70	70	70
*** His:Lys***	36	36	36	36	35	36	36	36	36	36	35
***NE, Mcal/kg***	2.59	2.54	2.48	2.58	2.59	2.48	2.43	2.38	2.48	2.48	2.45
***SID Lys:NE, g/Mcal***	5.26	5.25	5.24	5.26	5.26	5.44	5.43	5.41	5.44	5.44	5.25
***CP, %***	21.1	20.6	20.2	21.0	21.0	21.5	21.0	20.6	21.5	21.5	20.0
***Lactose, %***	15.00	14.63	14.25	15.00	15.00	7.50	7.31	7.13	7.50	7.50	—
***Ca, %***	0.49	0.48	0.46	0.49	0.49	0.57	0.56	0.55	0.57	0.57	0.64
***P, %***	0.52	0.51	0.50	0.52	0.52	0.55	0.54	0.53	0.55	0.55	0.56
***STTD P, %^i^***	0.47	0.46	0.45	0.47	0.47	0.46	0.45	0.45	0.46	0.47	0.43
***Crude fiber, %***	2.20	3.52	4.84	3.51	4.81	2.48	3.79	5.10	3.79	5.10	2.44
***NDF, %^j^***	7.49	9.18	10.88	9.04	10.58	8.25	9.93	11.60	9.80	11.34	8.51
***ADF, %^k^***	3.10	4.56	6.01	4.54	5.97	3.49	4.93	6.38	4.93	6.36	3.45
***Lignin, %***	0.42	0.89	1.37	0.89	1.37	0.51	0.98	1.45	0.98	1.46	0.53
***Analyzed composition***											
*** CP, %***	19.7	19.4	19.0	20.1	20.2	19.9	19.8	19.2	20.8	20.2	20.1
*** NDF, %***	6.6	7.6	9.4	9.6	14.3	6.1	8.3	10.4	8.8	9.7	8.2
*** ADF, %***	2.2	3.4	5.0	9.7	4.9	2.2	3.8	5.5	4.1	5.4	3.2
*** ADL, %^l^***	0.04	0.56	1.18	0.60	1.16	0.04	0.62	1.19	0.50	1.25	0.06
*** ABC-4, meq/kg^m^***	204	195	192	203	199	256	252	258	256	260	—

a Phases 1, 2, and 3 were fed for 10, 13, and 22 d, respectively. Diets were formulated to meet or exceed the nutrient requirements recommended by the NRC (2012) for pigs with average body weights of 6, 9, and 18 kg for phases 1, 2, and 3, respectively.

b ME-PRO (Aquatech, Brookings, SD).

c ValoproWin (Mixscience, Bruz, France).

d Provided per kilogram of feed: 4134 IU of vitamin A; 1653 IU of vitamin D; 44 IU of vitamin E; 3.31 mg of vitamin K3; 33 µg of vitamin B_12_; 8 mg of riboflavin; 28 mg of pantothenic acid; 50 mg of niacin.

e Provided per kilogram of feed: 110 mg of Zn as zinc sulfate; 17 mg of Cu as copper sulfate; 33 mg of Mn as manganese oxide; 110 mg of Fe as ferrous sulfate; 0.30 mg of I as ethylenediamine dihydriodide; 0.30 mg of Se as sodium selenite.

f Ronozyme Hiphos 2400 (dsm-firmenich, Parsippany, NJ) provided 2000 FYT/kg for phases 1 and 2 and 811 FYT/kg in phase 3 with an assumed release of 0.14% STTD P in phases 1 and 2 and 0.12% STTD P in phase 3.

g Primary Products Ingredients Americas LLC, Decatur, IL.

h Phormasil NA (Hawkins, Roseville, MN).

i Standardized total tract digestible phosphorus.

j Neutral detergent fiber.

k Acid detergent fiber.

l Acid detergent lignin.

m Acid binding capacity to a pH of 4.

For all experiments, experimental diets were manufactured at the Kansas State University O.H. Kruse Feed Technology Innovation Center in Manhattan, KS. The first two phases were fed in pellet form (pellet diameter 4.4 mm, 12% of fines, and 92% of PDI), and the common phase 3 diet in meal form. Feed samples by phase (approximately 2 kg) were collected during the bagging process. Samples were collected from every other bag for phases 1 and 2 and from every fifth bag for phase 3.

### Chemical analysis

Feed samples were analyzed in duplicate for crude protein (CP; Nitrogen × 6.25) using the Dumas combustion method (AOAC 2006; Method 990.03) with a LECO TruMac N analyzer (LECO Corporation., St. Joseph, MI). Neutral detergent fiber, ADF, and acid lignin were determined sequentially according to the procedures of Van Soest et al. (1991) using an Ankom Fiber Analyzer (Ankom Technology, Macedon, NY). Water-holding capacity and particle size of VLPW were analyzed in triplicated using the methods described by Ogunwolu et al. (2009) and Kalivoda et al. (2017), respectively.

Fecal samples were stored at 4°C. Samples were dried at 55°C for 48 h and weight loss was used to determine the percentage of fecal DM.

### Statistical analysis

All experiments were analyzed using RStudio [Version 4.0.2 (2020-06-22), R Core Team, R Foundation for Statistical Computing, Vienna, Austria]. All experiments were analyzed using a generalized randomized block design as experimental design with pen serving as the experimental unit. For Exp. 1 and 3 a factorial treatment structure was used, while in Exp. 2, treatments were randomly assigned within each BW Block. The performance data model utilized dietary treatment as a fixed effect and the BW block as a random effect. Barn also served as a random effect in experiments 1 and 2. In Exp. 1, contrast coefficients were set to evaluate the main effect of VLPW feeding duration (0, 10, or 24 d), formulation strategy (low ABC-4 diets or conventional with and without the inclusion of ZnO), and their interaction.

In Exp. 2, linear and quadratic effects of increasing dietary VLPW on performance and fecal dry matter were evaluated for the low ABC-4 diets. Specific contrasts compared the low- ABC-4 diets and conventional with no added VLPW.

In Exp. 3, contrast coefficients were set to evaluate the main effect of VLPW inclusion (2.5 or 5%), formulation method (diluted or adjusted), and their interaction. The linear and quadratic effects of increasing VLPW (0, 2.5, or 5%) within each formulation method on performance and fecal DM were evaluated. Contrast coefficients were established based on added VLPW level.

For all experiments, fecal dry DM was analyzed as repeated measurements, accounting for the two sampling days. In addition to the performance criteria included in each model within each experiment, pen was included as a random effect to account for subsampling associated with multiple individual pigs analyzed from each pen. Results were considered significant at *P* ≤ 0.05 and marginally significant at 0.05 < *P* ≤ 0.10.

## Results

### Experiment 1

The CP, NDF, ADF, and lignin of experimental diets were consistent with formulated values, considering analytical variation. No interactions were observed between VLPW feeding duration and formulation strategy on growth performance and fecal DM (Table 4). For the main effect of VLPW feeding duration, there were no differences observed between treatments for any of the performance criteria or periods. However, pigs fed diets containing 2.5% VLPW had greater (*P* = 0.019) fecal DM on d 10 than those fed diets without VLPW. For d 24 fecal DM, no differences between VLPW feeding duration were observed.

**Table 4:** Effects of ValoproWin feeding duration and formulation strategy on nursery performance and fecal dry matter, Exp. 1.^a^

	ValoproWin feeding duration, d^b^						Formulation strategy^c^			
						ABC-4:	Low ABC-4	Conventional		
Item	0	10	24	SEM	*P* =	ZnO:	No	Yes	SEM	*P* =
***BW, kg***										
*** d 0***	5.6	5.6	5.6	0.47	0.998		5.6	5.6	0.47	0.957
*** d 10^d^***	7.0	7.1		0.53	0.267		7.0	7.1	0.53	0.030
*** d 24***	12.4	12.3	12.4	0.86	0.901		12.0	12.8	0.85	< 0.001
*** d 42***	23.2	23.2	23.4	1.35	0.883		23.1	23.4	1.34	0.332
***Phase 1 (d 0 to 10)^d^***										
*** ADG, g***	145	153		11.8	0.276		141	155	11.4	0.033
*** ADFI, g***	172	180		7.7	0.163		166	183	7.3	0.001
*** G:F, g/kg***	845	839		43.9	0.784		847	840	42.1	0.821
***Phase 2 (d 10 to 24)***										
*** ADG, g***	380	376	386	25.4	0.726		358	403	24.9	< 0.001
*** ADFI, g***	497	479	503	29.8	0.268		463	523	29.1	< 0.001
*** G:F, g/kg***	767	783	766	16.9	0.469		775	769	15.6	0.674
***Experimental period (d 0 to 24)***										
*** ADG, g***	286	280	285	17.4	0.808		267	300	16.9	< 0.001
*** ADFI, g***	365	351	365	20.1	0.316		339	381	19.7	< 0.001
*** G:F, g/kg***	782	797	780	20.9	0.375		789	784	20.2	0.596
***Common diet (d 24 to 42)***										
*** ADG, g***	598	603	608	28.8	0.795		616	591	28.0	0.059
*** ADFI, g***	866	854	869	43.6	0.739		875	850	42.8	0.134
*** G:F, g/kg***	692	708	701	9.8	0.262		704	697	9.0	0.365
***Overall (d 0 to 42)***										
*** ADG, g***	419	418	424	21.8	0.847		417	424	21.4	0.404
*** ADFI, g***	579	566	581	29.9	0.395		569	582	29.5	0.190
*** G:F, g/kg***	725	739	729	12.4	0.147		733	729	12.0	0.535
***Fecal dry matter, %^e^***										
*** d 10^d^***	21.0	23.2		0.73	0.019		22.3	22.6	0.60	0.753
*** d 24***	22.5	22.3	22.2	0.59	0.952		23.3	21.4	0.51	0.004

a A total of 300 pigs (Line 241 × 600, DNA, Columbus, NE) were used in a 42-d growth study. No interactions between VLPW feeding duration and formulation strategy were observed for growth performance criteria or fecal dry matter in any period.

b ValoproWin (MiXscience, Bruz, France) is a fiber ingredient that combines a purified source of fiber with oat hulls and yeast autolysate. ValoproWin was fed for 0, 10, or 24 d. Diets containing ValoproWin were not reformulated to maintain nutrient concentrations; therefore, inclusion of ValoproWin resulted in an approximate 2.5% dilution of all dietary nutrients.

c Low ABC-4 diets were formulated to 200 and 250 meq/kg from d 0 to 10 and d 10 to 24, respectively. The lactose levels were reached using pure lactose, AX3 digest as a protein concentrate source, and fumaric and formic acid as acidifiers. The conventional diets were formulated to 493 and 470 meq/kg, containing 2990 and 1910 mg/kg of Zn from ZnO from d 0 to 10 and 10 to 24, respectively. Spray-dried milk whey powder and HP300 were used as lactose and specialty soy sources, respectively.

d For d 10 BW, period 1 performance, and fecal DM on d 10 only one value is presented for 10 and 24 d VLPW feeding duration because all pigs received the same diet during this period. The *P*-value tests diets with and without VLPW.

e Same three pigs per pen were sampled on d 10 and 24.

For the main effect of diet formulation strategy, during phase 1 (d 0 to 10), phase 2 (d 10 to 24) and the experimental period (d 0 to 24), pigs fed conventional diets with ZnO had greater (*P* ≤ 0.05) d 10 and 24 BW, ADG, and ADFI than those fed low ABC-4 diets without ZnO. However, no differences were observed for G:F between both formulation strategies. When transitioned to a common phase 3 diet (d 24 to 42), pigs previously fed diets with low ABC-4 without ZnO tended (*P* = 0.059) to have increased ADG; however, no differences were observed for ADFI and G:F. Overall (d 0 to 42), no differences were observed between the two formulation strategies for any of the performance criteria. There was no difference in fecal DM between formulation strategies on d 10; however, on d 24, pigs fed low ABC-4 diets without ZnO had greater (*P* = 0.004) fecal DM than those fed conventional diets with ZnO.

### Experiment 2

The CP, NDF, and ADF of experimental diets were consistent with formulated values, considering analytical variation (Table 5).

**Table 5:** Analyzed composition of experimental diets, Exp. 2 (as-fed basis).^a^

	Low ABC-4^b^					Conventional
*VLPW, %:^c^*	0.00	1.75	2.5	3.75	5.00	- - -
***Phase 1***						
*** CP, %***	20.6	19.9	19.9	19.6	19.5	19.8
*** NDF, %^e^***	8.1	8.5	9.8	8.7	12.7	6.6
*** ADF, %^f^***	2.2	2.6	2.9	3.6	3.2	2.6
*** ADL, %^g^***	0.03	0.28	0.54	0.86	1.22	0.05
*** ABC-4, meq/kg***	206	203	198	210	193	451
***Phase 2***						
*** CP, %***	21.1	21.0	20.9	19.7	19.4	21.1
*** NDF, %***	7.6	7.6	7.8	9.9	10.7	5.7
*** ADF, %***	2.2	2.8	3.3	3.2	3.6	2.8
*** ADL, %***	0.04	0.22	0.58	0.82	1.19	0.04
*** ABC-4, meq/kg***	253	255	260	259	264	448

a The values represent the mean of two samples. Phase 1 was fed for 10 d and phase 2 for 14 d in pellet form.

b Acid binding capacity at a pH of 4.

c ValoproWin (Mixscience, Bruz, France).

d Crude protein.

e Neutral detergent fiber.

f Acid detergent fiber.

g Acid detergent lignin.

For phase 1 (d 0 to 10), increasing VLPW decreased d 10 BW (linear, *P* = 0.033) and ADG (linear, *P* = 0.019; Table 6). There were no differences in ADFI. As a result, G: F decreased (linear, *P* = 0.003) as VLPW increased. No differences in d 10 BW, ADG, and ADFI were observed between the low ABC-4 and conventional diets. However, pigs fed low ABC-4 diets had increased (*P* = 0.007) G:F ratio.

**Table 6:** Effects of increasing ValoproWin (VLPW) in low ABC-4 diets, formulated without nutrient adjustment, on performance and fecal dry matter of nursery pigs, Exp 2.^a^

	Low ABC-4^b^					Conventional		*P* =^d^		
Item VLPW, %:^c^	0.00	1.75	2.50	3.75	5.00	0	SEM	Linear	Quadratic	ABC-4
***BW, kg***										
*** d 0***	6.3	6.3	6.3	6.3	6.3	6.3	0.26	0.933	0.960	0.893
*** d 10***	7.9	7.9	7.6	7.7	7.7	7.8	0.32	0.033	0.123	0.513
*** d 24***	13.6	13.4	12.9	13.0	13.3	13.6	0.44	0.160	0.049	0.948
*** d 42***	25.5	25.4	24.6	25.0	25.4	25.2	0.65	0.535	0.083	0.494
***Period 1 (d 0 to 10)***										
*** ADG, g***	159	155	133	138	142	152	8.6	0.019	0.084	0.441
*** ADFI, g***	174	177	155	165	170	183	7.9	0.302	0.114	0.310
*** G:F, g/kg***	914	877	852	830	835	832	20.9	0.003	0.259	0.007
***Period 2 (d 10 to 24)***										
*** ADG, g***	408	397	380	378	401	423	13.2	0.379	0.055	0.348
*** ADFI, g***	545	558	534	523	539	590	15.8	0.291	0.701	0.025
*** G:F, g/kg***	747	712	711	725	744	717	12.6	0.855	0.007	0.076
***Experimental period (d 0 to 24)***										
*** ADG, g***	304	296	277	278	293	310	9.5	0.120	0.029	0.621
*** ADFI, g***	391	399	376	374	385	420	11.1	0.229	0.439	0.028
*** G:F, g/kg***	778	742	735	745	761	738	10.5	0.348	0.004	0.010
***Common period (d 24 to 42)***										
*** ADG, g***	660	663	646	666	667	645	13.9	0.603	0.425	0.322
*** ADFI, g***	1056	1046	1023	1042	1037	1021	22.7	0.428	0.407	0.149
*** G:F, g/kg***	626	634	633	639	645	632	7.8	0.091	0.981	0.580
***Overall (d 0 to 42)***										
*** ADG, g***	457	454	435	445	454	452	10.2	0.515	0.067	0.666
*** ADFI, g***	676	676	653	660	665	675	14.3	0.224	0.310	0.961
*** G:F, g/kg***	676	670	667	673	683	670	6.7	0.437	0.103	0.497
***Fecal dry matter, %^e^***										
*** d 10***	26.9	25.8	27.9	27.8	29.0	24.3	0.87	0.027	0.464	0.039
*** d 24***	24.4	23.9	26.7	26.4	26.6	23.5	0.87	0.018	0.612	0.466

a A total of 360 pigs (Line 200 × 400, DNA, Columbus, NE) were used in a 42-d growth study with five pigs per pen and 12 pens per treatment.

b ABC-4, Acid binding capacity to a pH of 4. Low ABC-4 diets were formulated to 200 and 250 meq/kg from d 0 to 10 and d 10 to 24, respectively, and did not contain pharmacological levels of Zn. High ABC-4 diets were formulated to 493 and 470 meq/kg, containing 2990 and 1910 mg/kg of Zn from ZnO from d 0 to 10 and 10 to 24, respectively.

c VLPW, ValoproWin (MiXscience, Bruz, France), is a fiber source that contains a purified source of fiber with oat hulls and yeast autolysate.

d Linear and quadratic *P*-values evaluated the effect of VLPW level within the low ABC-4 diets, whereas the ABC-4 *P*-value compared the low and high ABC-4 diets when VLPW was not added. Diets containing ValoproWin were not reformulated to maintain nutrient concentrations; therefore, inclusion of ValoproWin resulted in dilution of all dietary nutrients proportional to its inclusion level.

e The same three pigs per pen were sampled on d 10 and 24. There was a significant effect of sampling day (*P* = 0.001); however, no significant interactions (*P* > 0.10) between day and the linear or quadratic effects of VLPW inclusion were observed.

For phase 2 (d 10 to 24), d 24 BW decreased (quadratic, *P* = 0.049) as VLPW increased with no change in BW between 0 and 1.75%, followed by a reduction at 2.50 and 3.75%, and returning to the control values with 5.0% VLPW. Average daily gain tended (quadratic, *P* = 0.055) to decrease as VLPW increased, where ADG decreased as VLPW increased from 0% to 3.75% and then returned to the control values at 5%. No response to increasing VLPW was observed for ADFI, and as a result, G:F decreased (quadratic, *P* = 0.007). The gain: feed ratio decreased between 0% and 2.5% followed by an increase between 3.75% and 5.0%. There were no differences between low ABC-4 and conventional with added ZnO diets in ADG. Pigs fed low ABC-4 diets had decreased ADFI (*P* = 0.025) compared with pigs fed conventional diets. As a result, low ABC-4 diets tended (*P* = 0.076) to increase G:F ratio.

For the experimental period (d 0 to 24), ADG decreased then increased (quadratic, *P* = 0.029) with increasing VLPW. Average daily gain decreased between 0% and 3.75% and then increased at 5%. No response to increasing VLPW was observed for ADFI. As a result, G:F decreased then increased (quadratic, *P* = 0.006) as VLPW increased, with G:F becoming poorer as VLPW increased from 0% to 2.50% followed by an improvement between 3.75% and 5.0%. There were no differences between pigs fed the low ABC-4 or conventional diets for ADG. However, pigs fed the low ABC-4 diets had decreased (*P* = 0.028) ADFI, resulting in increased (*P* = 0.010) G:F compared to those fed the conventional diets.

For the common period (d 24 to 42), no response was observed in pigs previously fed VLPW during the experimental period for ADG and ADFI. However, G:F tended (linear, *P* = 0.091) to increase as VLPW increased in phase 1 and 2 diets. No differences between pigs previously fed low ABC-4 or conventional diets were observed for any of the performance criteria.

Overall (d 0 to 42), increasing VLPW during the experimental period (d 0 to 24) tended to decrease then increase 42 BW (quadratic, *P* = 0.083), where BW decreased as VLPW increased from 0% to 3.75% in phases 1 and 2 and then returned to the control values at 5.0%. Similarly, ADG tended to decrease then increase (quadratic, *P* = 0.067), with the lowest ADG observed in pigs fed 2.50% and returned to the control values at 5.0%. No responses were observed for ADFI and G:F. No differences in pigs fed low ABC-4 or conventional diets were observed for any performance criteria.

On d 10 and 24, fecal DM increased (linear, *P* < 0.005) with increasing VLPW. Pigs fed low ABC-4 diets had increased (*P* = 0.039) fecal DM on d 10 compared with hose fed the conventional diets; however, no differences were observed between low ABC-4 or conventional diets on d 24 fecal DM.

### Experiment 3

The CP, NDF, ADF, and lignin of experimental diets were consistent with formulated values, considering analytical variation (Table 3). No VLPW and formulation method interactions were observed for any of the performance criteria (Table 7). For phase 1 (d 0 to 10), no VLPW or formulation method main effects were observed for any of the performance criteria. Similarly, no linear or quadratic interactions or main effects were observed for any of the performance criteria with increasing VLPW.

**Table 7:** Effects of ValoProWin (VLPW) and diet formulation method on growth performance and fecal dry matter in nursery pigs, Exp. 3.^a^

		Diluted^b^		**Adjusted**			*P* =^d^		
Item VLPW, %:^c^	0	2.5	5.0	2.5	5.0	SEM	Formulation × VLPW	Formulation	VLPW
***BW, kg***									
*** d 0***	5.6	5.6	5.6	5.6	5.6	0.87	0.968	0.856	0.676
*** d 10***	6.5	6.6	6.5	6.4	6.3	1.01	0.829	0.163	0.347
*** d 23***	12.2	12.5	12.1	12.0	12.0	1.68	0.299	0.203	0.485
*** d 45***	26.8	27.1	27.4	26.7	26.8	2.76	0.806	0.213	0.726
***Period 1 (d 0 to 10)***									
*** ADG, g***	93	100	91	88	74	16.7	0.818	0.130	0.208
*** ADFI, g***	125	129	131	126	112	17.9	0.341	0.175	0.470
*** G:F, g/kg***	636	756	610	686	644	77.4	0.502	0.816	0.230
***Period 2 (d 10 to 23)***									
*** ADG, g***	432	447	431	425	440	51.6	0.132	0.526	0.973
*** ADFI, g***	511	529	521	491	507	59.9	0.349	0.043	0.735
*** G:F, g/kg***	851	846	829	866	867	11.2	0.431	0.011	0.466
***Experimental period (d 0 to 23)***									
*** ADG, g***	283	293	281	279	281	35.8	0.429	0.402	0.605
*** ADFI, g***	342	352	350	332	335	41.2	0.807	0.091	0.936
*** G:F, g/kg***	829	834	804	838	836	9.6	0.135	0.054	0.088
***Period 3 (d 24 to 45)***									
*** ADG, g***	668	667	694	671	670	49.4	0.202	0.370	0.250
*** ADFI, g***	995	995	1040	1001	1032	81.8	0.723	0.950	0.063
*** G:F, g/kg***	671	673	670	672	652	9.0	0.326	0.243	0.168
***Overall (d 0 to 45)***									
*** ADG, g***	470	474	482	471	471	42.2	0.667	0.392	0.622
*** ADFI, g***	660	663	686	659	676	60.6	0.836	0.578	0.137
*** G:F, g/kg***	712	717	704	714	698	7.3	0.809	0.552	0.057
***Fecal dry matter, %^e^***									
*** d 10^f^***	22.0	25.1	26.0	25.4	26.8	1.38	0.753	0.516	0.192
*** d 24^g^***	23.2	24.2	25.2	23.5	25.3	1.38	0.619	0.718	0.123

a A total of 335 pigs (Line 241 × 600, DNA, Columbus, NE) were used in a 45-d growth study with four or five pigs per pen and 14 replicate pens per treatment. Dietary treatments were assigned in a 2 × 2 + 1 factorial experiment, with main effects of VLPW inclusion (2.5 or 5.0%) and formulation method (diluted or adjusted), and an extra treatment was included where no VLPW was included in the formulation.

b In the diluted formulation method, the VLPW was included without any nutritional adjustments in the diet, which resulted in a dilution of all nutrients as VLPW increased. In the adjusted formulation method, the chemical composition of the diet was adjusted to the values of the diets without VLPW.

c VLPW, ValoproWin (MiXscience, Bruz, France), is a fiber source that contains a purified source of fiber with oat hulls and yeast autolysate.

d The *P*-value associated with formulation compares the diluted and adjusted formulation method when VLPW was included at 2.5 or 5.0%. Linear and quadratic effects of increasing VLPW (0, 2.5, or 5%) within each formulation method, and their interaction, were evaluated. No linear, quadratic, or interaction effects were detected for most performance criteria. However, during the experimental period, a tendency for a linear interaction (*P* = 0.087) was observed for G: F, where G: F tended to linearly increase (*P* = 0.055) with increasing VLPW in the diluted diets but not in the adjusted diets. In period 3, ADG tended to linearly increase (*P* = 0.097) with increasing VLPW in the diluted formulation method.

e Fecal samples were collected from the same 3 pigs per pen on d 10 and 23 No interactions between sampling day and dietary treatment or significant main effect of sampling day were observed. Linear and quadratic effects of increasing the VLPW (0, 2.5, or 5%) within each formulation method were evaluated.

f On d 10 fecal DM, increased (linear, *P* < 0.001) as the VLPW increased, independent of the formulation method.

g On d 24, Fecal DM tended to increase (linear, *P* = 0.098) as VLPW increased in the adjusted formulation method.

For phase 2 (d 10 to 23), pigs fed the diluted diets had greater ADFI (*P* = 0.043) than those fed adjusted diets. However, no differences were observed in d 23 BW and ADG between formulation method. As a result, G:F increased (*P* = 0.011) in pigs fed the adjusted diets compared with those fed diluted diets. No main effects of increasing VLPW were observed for any of the performance criteria during this period. Similarly, no linear or quadratic interactions or main effects were observed for any of the performance criteria as VLPW increased.

For the experimental period (d 0 to 23), pigs fed diluted diets tended (*P* = 0.091) to have greater ADFI than those fed adjusted diets. No differences were observed in ADG between formulation methods. Consequently, pigs fed adjusted diets tended (*P* = 0.084) to have greater G:F than those fed diluted diets. No VLPW main effects were observed for ADG and ADFI; however, pigs fed diets containing 5% VLPW tended (*P* = 0.054) to have decreased G:F compared with those fed diets containing 2.5% VLPW. Increasing the VLPW resulted in no linear or quadratic interactions or main effects for d 23 BW, ADG, and ADFI. However, a tendency for a linear interaction (*P* = 0.087) was observed for G:F, where G:F tended to linearly decrease (*P* = 0.055) as VLPW increased in diluted diets but not in the adjusted diets.

When pigs were fed a common diet during phase 3 (d 23 to 45), no main effects of formulation method were observed for any of the performance criteria. Pigs previously fed diets with 5% VLPW during the experimental period tended (*P* = 0.063) to have greater ADFI than those fed 2.5% VLPW; however, no effect of VLPW level was observed for d 45 BW, ADG, or G:F. Increasing VLPW during the experimental period resulted in no linear or quadratic interactions or main effects for ADFI and G:F during the common phase. However, despite the lack of interactions, ADG tended to increase (linear, *P* = 0.097) as VLPW increased during the experimental period in diluted diets but not in adjusted diets.

For the overall period (d 0 to 45), no main effect of formulation method was observed for any performance criteria. Pigs fed diets with 5% VLPW during the experimental period tended (*P* = 0.057) to have decreased G:F compared with those fed 2.5% VLPW; however, no effect of VLPW level was observed for ADG and ADFI. No linear or quadratic effects were observed for any of the performance criteria with increasing VLPW during the experimental period.

For fecal DM, no interactions between sampling day and dietary treatment or a significant main effect of the sampling day were observed. Similarly, no interactions or main effects of formulation method or increasing VLPW were observed for d 10 and 23 fecal DM. However, increasing VLPW increased (linear, *P* < 0.001) fecal DM on d 10, independent of formulation method. However, on d 24, fecal DM tended to increase (linear, *P* = 0.098) with increasing VLPW in adjusted diets but not in diluted diets.

## Discussion

The addition of fiber-rich ingredients in nursery pig diets has emerged as a nutritional approach to enhance intestinal structure and function by stimulating gut motility, modulating microbiota composition, and improving gut barrier function (Agyekum and Nyachoti 2017). However, these positive effects do not always result in improvements in growth performance, possibly due to the complex interactions between fiber type, physicochemical properties, formulation strategy, and dietary composition, as well as intrinsic animal factors such as age, health status, and genetics (Agyekum and Nyachoti 2017; Berrocoso et al. 2015; Hu et al. 2023).

The inclusion of fiber-rich ingredients in nursery diets without adjusting nutrient composition has been previously reported (Batson et al. 2021b; Laskoski et al. 2021). This approach is based on the assumption that pigs regulate their voluntary feed intake based on energy intake (NRC 2012). Therefore, reducing dietary energy density through the inclusion of fibrous ingredients may stimulate voluntary feed intake, thereby increasing the intake of nutrients other than energy. While adding fiber is a strategy to promote feed intake and overall performance in nursery pigs, other nutritional strategies have also been proposed to optimize pig growth and gut health. Among these, acidification of the gastrointestinal tract has been proposed to optimize nutrient utilization (Partanen and Mroz 1999) and reduce colonization by pathogenic bacteria (Ravindran and Kornegay 1993). However, it remains unclear whether an additive effect exists when combining acidification with other nutritional strategies. Therefore, the present series of experiments evaluated the interactions between dietary acidification and fiber, using VLPW as the dietary fiber source.

Experiment 1 was designed to evaluate the effects of VLPW feeding duration and low ABC-4 compared with a more conventional formulation strategy (with pharmacological additions of Zn) on growth performance and fecal DM using the manufacturer’s recommended inclusion rate (2.5%). The results indicated that for growth performance, there was no interaction between formulation strategy and VLPW feeding duration, and contrary to what was expected, the use of VLPW did not influence growth performance. These findings suggest that 2.5% VLPW in corn–soybean meal-based diets may not have been enough to promote feed intake and improve growth performance. Consequently, Exp. 2 was conducted to evaluate increasing levels of VLPW in low ABC-4 diets up to 5%. Based on manufacturer recommendations and MixScience internal economic analysis. Contrary to expectations, performance responses were negative, particularly during the experimental period, suggesting that added fiber without maintaining the main nutrient concentration (controlled amino acids, energy, and macro-minerals) may have decreased nutrient intake and/or decreased nutrient digestibility. Given these results, it was hypothesized that formulation method could influence the response to added fiber. Therefore, Exp. 3 was designed to evaluate whether adjusting diets to account for the dilution effect of VLPW would mitigate the negative effects previously observed.

In European-type diets based on wheat and barley, without the use of pharmacological levels of minerals and with low CP levels, the inclusion of insoluble fiber (a combination of wheat straw and oat hulls or lignocellulose) has shown positive effects on growth performance (Gerritsen et al. 2012; Superchi et al. 2017). Furthermore, specific insoluble fiber sources have been shown to directly enhance gut health; for instance, the inclusion of wheat bran increases short-chain fatty acid concentrations and reduces Escherichia coli counts in the gastrointestinal tract (Molist et al. 2011), while the addition of a coarse, indigestible plant fiber concentrate modulates gut microbiota activity and reduces post-weaning diarrhea scores (Lemoine et al. 2022). The beneficial response in these feeding systems may be attributed to the higher supply of fermentable non-starch polysaccharides from ingredients such as wheat and barley (NRC 2012), which, in conjunction with the insoluble sources, provide adequate substrate for beneficial intestinal bacteria (Chen et al. 2020) and the production of beneficial metabolites such as volatile fatty acids (Jha and Leterme 2012). However, when applied to corn-soybean meal-based diets, positive responses of insoluble fiber on performance and gut health are less frequently observed. This discrepancy is likely due to differences in fiber type and fermentability, as corn or soybean meal-derived fiber is primarily insoluble and less fermentable than fiber fractions from small grains (Jha et al. 2019; Lv et al. 2022).

The lack of a positive growth response to VLPW on growth performance in the present experiments may also be attributed to the ratio of soluble to insoluble fiber and the health status of the pigs. Insoluble fiber can increase digesta passage rate and reduce nutrient digestibility when not properly balanced with fermentable fractions, potentially limiting the anticipated benefits on health and growth performance (Acosta et al. 2020; Jha and Berrocoso 2015). Additionally, nursery pigs in high-health production systems, such as those used in the current experiments, may benefit less from fiber supplementation compared with pigs reared under health-challenging conditions (Berrocoso et al. 2015) where fiber can contribute to stabilizing gut microbiota and reducing pathogen colonization (Pluske et al. 2018). Collectively, these observations underscore the importance of optimizing the balance between soluble and insoluble fiber in nursery diets and highlight the need for further research to determine the optimal fiber composition under varying formulation strategies and farm health conditions.

Despite the absence of published information directly evaluating VLPW in nursery diets, comparisons of the current results can be made with studies assessing the effect of insoluble fiber and yeast autolysate on nursery pig performance. Similar to the results observed in Exp. 1, others have reported a lack of significant responses to increasing levels of insoluble fiber in corn-soybean meal-based diets on nursery pig performance. Batson et al. (2021a) indicated that the addition of 4% wheat middlings in corn–soybean meal-based nursery diets increased dietary NDF from 4.6% to 6.0% in phase 1 and from 6.0% to 7.2% in phase 2 but did not affect growth performance in any of the phases compared with corn–soybean meal diets formulated without pharmacological levels of Zn. Similarly, Laskoski et al. (2021) observed that the addition of 4% coarse wheat bran in corn–soybean meal-based nursery diets without pharmacological levels of Zn did not influence growth performance. Also, Batson et al. (2021b) observed no changes in performance associated with the addition of three fiber sources (4% coarse wheat bran, 1.85% oat hulls, or 1.55% cellulose) in corn-soybean meal-based diets. Collectively, these findings suggest that in corn-soybean meal-based diets, moderate additions of insoluble fiber, such as those evaluated in Exp. 1, may not affect nursery pig growth performance.

In growing pigs fed corn–soybean meal-based diets, Petry et al. (2020) observed a decrease in ADG and G:F with diets containing 30% corn bran without solubles, which increased the NDF content of the diet from 7.54% to 21.91%. Although the NDF levels evaluated in that experiment are not directly comparable with those tested in the present studies, the observed decrease in growth performance underscores the potential negative consequences of excessive insoluble fiber inclusion, which is mainly related to a dilution effect of dietary energy and/or reduction in nutrients digestibility. Similarly, De Jong et al. (2014) observed that increasing NDF concentration of nursery corn–soybean meal-based diets from 8.1% to 11.6% by increasing wheat middlings resulted in a linear reduction in ADG and ADFI, whereas no effect was observed on G:F. Although the NDF concentrations in that study were similar to those evaluated in Exp. 2 of the present project, the quadratic responses for ADG and G:F observed herein differ from the linear effects observed by De Jong et al. (2014). This discrepancy may be attributed to differences in the chemical composition of the fiber sources, as the addition of VLPW in the current experiment likely contributed additional nutritional factors beyond fiber, which supported improved performance.

The increased ADFI observed during the experimental period in Exp. 3, especially in the diluted diets, was expected, as in pigs, voluntary feed intake is largely regulated by dietary energy, meaning that when dietary energy is reduced, pigs typically increase feed intake to maintain a constant energy intake (NRC 2012). In contrast to the present results, previous studies have reported improvements in ADG and feed efficiency when fiber ingredients are added to diets formulated to maintain the same energy density as control diets (Gasa et al. 2010; Molist et al. 2011; Zhao et al. 2018). Such improvements may be related to enhanced intestinal health, which could reduce nutrient demands for immune function, thereby allowing greater nutrient utilization available for growth.

Autolyzed yeast (*Saccharomyces cerevisiae*) is one of the components in VLPW, and has been observed to improve growth performance in nursery pigs through mechanisms such as enhanced gut health, immune modulation, and nutrient absorption. Barducci et al. (2024) observed that replacing blood plasma with autolyzed yeast, with or without immunomodulators such as β-glucans, improved feed efficiency and reduced the need for feed-grade medications. Similarly, Correia et al. (2024) observed that added autolyzed yeast increased ADG and G:F by improving intestinal barrier integrity and upregulating the expression of nutrient transporters and antioxidant enzymes in the jejunum. Namted et al. (2022) also observed positive effects of added autolyzed yeast on both growth performance and gut health in weaned pigs, likely attributed to bioactive components such as β-glucans and mannan-oligosaccharides. Collectively, these findings suggest that autolyzed yeast is a viable nutritional strategy to enhance growth performance and intestinal health in nursery pigs. However, the lack of responses observed in the present experiments may indicate potential interactions between fiber and autolyzed yeast that attenuated the response, or that the concentration of autolyzed yeast in the VLPW product was insufficient to elicit measurable improvements in growth performance.

The consistent positive response of VLPW on fecal dry matter was expected, as insoluble fiber fractions such as cellulose and hemicellulose increase the WHC of digesta through their physicochemical properties and structural matrix formation. By retaining water within the fiber-digesta complex, insoluble fibers reduce the proportion of free luminal water available for excretion, resulting in firmer fecal consistency and increased fecal dry matter content (Jha and Berrocoso 2015; Wilfart et al. 2007). Recent work in growing pigs has shown that diets enriched with insoluble fiber sources modify digesta transit and water distribution along the gastrointestinal tract, supporting a shift toward greater fecal bulk density and reduced free water excretion (Dorado-Montenegro et al. 2023). In addition, fiber physicochemical characteristics, including swelling capacity and hydration properties, have been shown to be key determinants of fecal physicochemical traits and water balance in swine, further linking WHC to fecal DM outcomes under practical feeding conditions (Crome et al. 2025). In addition, the increased bulk accelerates digesta passage rate, which may reduce the proliferation of pathogenic microbiome in the gastrointestinal tract by limiting their colonization time and substrate availability (Canibe et al. 2022). This mechanical effect is complemented by a greater microbiome diversity and stimulation of hindgut fermentation (Yong et al. 2025), which together creates optimal gastrointestinal conditions that promote intestinal health, resulting in greater fecal DM. Similar to the present study, an improvement in fecal DM associated with the use of insoluble dietary fiber (Batson et al. 2021b; Chen et al. 2020) and autolyzed yeast (Lee et al. 2021) in nursery pigs have been previously reported.

Although improvements in fecal DM are not directly associated with enhanced growth performance in nursery pigs, greater fecal DM may still have important implications under commercial production conditions. In systems where labor costs and availability are constant constraints and pigs are exposed to greater enteric health challenges, increased fecal DM can result in cleaner piglets, which may reduce the need for therapeutic interventions and subsequently lower medication and labor costs. Furthermore, cleaner facility pens reduce the time and water required for sanitation between batches. However, in the present experiment, pigs were maintained under good health conditions and the use of injectable antibiotics was minimal; therefore, it was not possible to quantify these potential economic benefits. These results highlight the need for further studies under commercial conditions to evaluate the economic impact of dietary strategies, such as the inclusion of insoluble fiber, on fecal consistency and associated production costs in nursery pigs.

As previously described, in all experiments, VLPW was added to low ABC-4 diets without pharmacological levels of Zn. In Exp. 1 and 2, the conventional diets formulated without consideration of ABC-4 contained pharmacological levels of Zn, indicating that differences in performance between the two formulation strategies reflect the combined effects of ABC-4 and pharmacological levels of Zn, making it impossible to isolate the individual contribution of each factor. When diets did not contain pharmacological levels of Zn, the use of low ABC-4 diets improved growth performance compared with non-controlled ABC-4 diets (Stas et al. 2025). However, these same authors reported that when pharmacological levels of Zn from ZnO were included with the use of low ABC-4 diet, the combination did not provide any performance advantage, which is consistent with the results from Exp. 1 and 2. The lack of response to low ABC-4 diets under these conditions may be associated with factors beyond modulation of the gastrointestinal microbiota or health status of the pigs.

Pharmacological levels of Zn have been shown to increase circulating ghrelin, a hormone that in its exogenous form stimulates feed intake and growth in pigs (De Mille et al. 2022). In addition, improvements in intestinal morphology, including greater villus height, have been reported with pharmacological Zn addition (Peng et al. 2019), which may further enhance nutrient utilization and growth performance. Despite the positive effects of conventional diets with pharmacological levels of Zn on growth performance during the experimental period, these responses were not maintained overall. This response is consistent with previous reports showing that the advantages of pharmacological levels of Zn tend to disappear once pigs transition to a common diet (Carlson et al. 1999; Stas et al. 2025). The reduction in performance may be associated with reductions in circulating ghrelin, as well as restructuring of the gastrointestinal microbiome, which together could contribute to a reduction in growth rate. Consistent with previous reports (Arroyave et al. 2026; Stas et al. 2025), low ABC-4 diets improved fecal DM, possibly by creating gastrointestinal conditions favorable for beneficial bacteria such as *Lactobacillus* spp., which can inhibit *E. coli* adhesion.

In conclusion, the use of VLPW during the first two dietary phases may serve as a nutritional strategy to consistently increase fecal DM in nursery pigs. Considering the results of the three experiments collectively, during the experimental period the inclusion of VLPW had no effect on ADG. However, during the same period the inclusion of VLPW without adjusting dietary chemical composition resulted in greater ADFI and G:F. These differences were diluted after the common feeding period, and overall, no differences in growth performance associated with the use of VLPW were observed. Overall, no differences in growth performance were observed between the low and conventional diets, however, pigs fed low ABC-4 diets had greater fecal DM than those fed the conventional diets.

## List of abbreviations

ABC-4: Acid binding capacity to a pH of 4
ADF: acid detergent fiber
ADG: average daily gain
ADFI: average daily feed intake
BW: body weight
DM: dry matter
Exp.: experiment
G: F: gain-to-feed ratio
NDF: neutral detergent fiber
SBM: soybean meal
VLPW: ValoproWin
WHC: water-holding capacity
